# Factors influencing development of pulmonary tuberculosis among TB patients in Osun State - A Case Control Study

**DOI:** 10.4314/ahs.v25i3.3

**Published:** 2025-09

**Authors:** Sunday Olakunle Olarewaju, Omoyele Oluwaseun Omotola, Oyediji Bolanle Racheal, Abosede Abolaji Olorunnisola, Adedokun Adeyinka, Oluwafikayo Adeyemi-Benson, Olashoore Emmanuel, Akinwale John Faniyi

**Affiliations:** 1 Department of Community Medicine, Osun State University, Osogbo, Nigeria; 2 Department of Kinesiology & Community Health, College of Applied Health Sciences, University of Illinois at Urban-Champaign, Urbana, IL, USA; 3 Strategic Information Advisor, USAID-TB DIAH/John Snow Inc; 4 Laboratory Department, International Foundation Against Infectious Disease in Nigeria

**Keywords:** HIV/AIDs, Pulmonary Tuberculosis, TB Patients

## Abstract

**Background information:**

Tuberculosis (TB) is a bacterial infection caused by Mycobacterium tuberculosis that primarily affects the lungs but can also affect other parts of the body. Pulmonary TB is an infectious disease caused by Mycobacterium tuberculosis, which manifests in the lungs.

**Methodology:**

A case-control study that uses multi-stage sampling technique was used to select 216 respondents (108 TB Patients and 108 non-TB Patients). Respondent were interviewed using pre-test semi structured self-administered questionnaires, univariate and bivariate analysis was done using Statistical Package for Social Sciences (SPSS) with P value set as P < 0.05

**Result:**

The mean age of cases is 43.9 years, while it is 42 years for controls. However, age does not show a statistically significant association with TB status. There are more males in both the cases (78.6%) and controls (78.6%). There are no significant differences in TB status between single, married, divorced, separated, and widowed individuals. Odd of having tuberculosis is influenced by cigarettes and alcohol bottles consumed per day (p = 0.009 for cigarettes and p = 0.122 for alcohol bottles), having HIV (P less than 0.05).

**Conclusion:**

This study concludes that the development of pulmonary tuberculosis is influenced by a range of factors, including lifestyle habits, comorbid conditions such as HIV/AIDs and being diabetics. Effective control requires collaboration among various stakeholders and lifestyle modification. Public health campaigns could also be used to raise awareness about the link between smoking and TB.

## Background

Tuberculosis (TB) is a bacterial infection caused by Mycobacterium tuberculosis that primarily affects the lungs^1^. It is spread through the air when an infected person coughs or sneezes and can be life-threatening if left untreated. Despite global efforts to control and prevent TB, the disease continues to be a major public health problem, particularly in low- and middle-income countries. In 2020, an estimated 10 million people fell ill with TB and 1.4 million died from the disease^2^. Pulmonary TB is an infectious disease caused by Mycobacterium tuberculosis, which manifests in the lungs. These mycobacteria are transmitted through airborne droplets from a patient with pulmonary TB^3^,^4^. Tuberculosis (TB) remains a major public health issue across the world. A study reported that tuberculosis is one of the major public health problems that is most frequently the cause of death among adults despite being nearly 100% curable. It has also affected almost one-third of the world's population. One of the challenges in controlling TB is the presence of resistance. In Nigeria, local evidence shows similar concerns; for instance, a study in Osun State reported a high prevalence and incidence of TB^5^,^6^.

Morbidity and mortality from tuberculosis have been on the increase in spite of increased understanding of its natural history and major advances in the technology involved in its management within the past 50 years. Indeed, Mycobacterium tuberculosis remains the world leading cause of death from a single infectious agent. Every year, about eight million people develop this disease and some three million dies of it, over 95% of these from developing countries such as Nigeria^7^. It is noteworthy that most of the deaths from developing countries occur in persons aged between 15 and 59 years, thereby making tuberculosis a leading cause of death within the economically productive groups of the population. Among the countries are India, Indonesia, Pakistan, Bangladesh, China and Nigeria, and progress in these high-incidence countries has been described as the key to global tuberculosis control^8^.

Nigeria has one of the highest burdens of TB in Africa. There were more than 84,000 new cases of TB in Nigeria in the year 2011 in TB programs in Nigeria which consistently yielding figures far below the WHO's 85% treatment success target. For instance, field surveys in Nigeria yielded TB treatment success rates ranging from 42% in South-South Nigeria, through 64-76% in South West to 40% in Northern Nigeria^9^.

Osun State in Nigeria has been identified as one of the high-burden areas for TB. Despite the efforts made by the government and international organizations to control the spread of TB, the incidence of the disease remains high in the state. There is a need to identify the factors that contribute to pulmonary TB in Osun State to inform the development of effective TB control and prevention strategies.^10^.

Approximately 75% of patients with pulmonary TB are in the economically productive age group (15-50 years). It is estimated that an adult with pulmonary TB will lose an average of 3-4 months of working time. The results in an annual loss of household income of around 20–30%. If a person dies from pulmonary TB, this person will lose income for about 15 years. Apart from being economically detrimental, pulmonary TB also has a negative social impact, one of which is being ostracized by the society^11^.

Furthermore, with the complexity of risk factors may development of pulmonary TB is critical for reducing the disease's incidence and impact. The need for targeted TB control and prevention strategies: By identifying the factors that contribute to the development of pulmonary TB, health authorities and communities can target interventions to populations at considerable risk of the disease. This can reduce the incidence and impact of TB in the region^12^.

The importance of understanding the impact of demographic, environmental, behavioral, and socioeconomic factors on the development of TB.

These factors are known to influence the incidence of TB (and understanding their impact on the development of pulmonary TB can inform the development of effective TB control and prevention strategies^13^.

Early diagnosis and effective treatment of TB can significantly reduce the transmission of the disease and improve patient outcomes. Understanding the factors that result in pulmonary TB can inform the development of early diagnostic tools and effective treatments for the disease. Therefore, this study examined the Factors Influencing Development of Pulmonary Tuberculosis Among Tb Patients in Osun State - A Case Control Study

## Materials and Methods

### Study Location

Osun state was created in 1991 from the eastern third of Oyo state. Of the 36 states of Nigeria, Osun is the ninth smallest in area and nineteenth most populous with an estimated population of about 4.7 million as of 2016.

### Study Design and Population

The study design adopted for this research project is a case-control study. In this case-control study, individuals with the disease of interest (cases) are compared to those without the disease (controls) to identify potential risk factors for the disease. This case-control study was conducted on a subset of the population diagnosed with pulmonary tuberculosis (TB) in Osun State, Nigeria.

### Sample Size Determination and Sampling Technique

The minimum sample size of 100 was calculated using The sample size determined for this study was calculated using the formula when the interest is to test a hypothesis comparing some exposure of two groups:

n = (Zα/2 + zβ)^2 * (p1 * (1-p1) + p2 * (1-p2)) / (p1 - p2)^2

The significance level (alpha): This is typically set at 0.05, which represents a 5% chance of making a type I error (rejecting the null hypothesis when it is true).

The power of the study (1-beta): This is typically set at 0.80, a 80% chance of detecting a true effect if one exists. The odds ratio (OR) or relative risk (RR): This is typically set at 2:0 (this represents the strength of the association between the exposure and outcome). n = sample size

Zα/2 = the critical value for the selected alpha level is (1.96, for alpha=0.05)
Zβ = the critical value for the selected power (0.84 for power=0.80)
p1 = proportion related to research objective from cases (TB patients with pulmonary tuberculosis)
p2 = proportion related to research objective among controls

In this case, the p1 = 0.275 (GJ Oyedeji 2020) (27.5% of cases have pulmonary tuberculosis) and p2 = 0.4 (40% of controls).

Plugging in the values, we get:mn = (1.96 + 0.84)^2 * ((0.275 * 0.4) + (0.4 * 0.275)) / (0.275- 0.4)^2 = 107.8 Rounding up to the nearest whole number, we need a sample size of 108 cases and 108 controls, for a total of 216 participants. Therefore, a total of 216 participants, consisting of 108 cases and 108 controls, would be needed for a case-control study on factors influencing the development of pulmonary tuberculosis among TB patients in Osun state.

Multi-stage sampling technique was used in the selection of the respondents.

### Research instrument and data collection methods

A structured interviewer-administered questionnaire was used as the study instrument. This was designed to seek information about factors Influencing Development of Pulmonary Tuberculosis Among Tb Patients in Osun State. Research assistants comprising of TB field workers supporting health facilities in the delivery of community TB screening and who were familiar with TB patients were engaged and trained before data collection.

### Validation and pre-test of the instrument

The questionnaire was pretested among eligible TB patients attending health facilities in Lagos state General Hospital, which was not among the selected facilities for the study. This helped to know whether the questionnaire assessed what it intended to measure and whether the language and organization were appropriate to address the objectives of the study. The responses provided also helped to address any form of ambiguity in the questionnaire as well as modified questions or response categories where necessary. The Cronbach's alpha coefficient of the questionnaire was 0.78, indicating an accept- able internal consistency.

### Measurement of outcome variables

The mean score of the maximum score for the responses were calculated. The respondents who scored below the mean were regarded as having poor knowledge or negative attitude

### Data Analysis

Questionnaires were sorted out to check for errors and omissions. Thereafter, data were entered into the computer and analyzed using Statistical Package for Social Sciences (SPSS) version 23. Frequency distribution tables, and graphs were generated from variables while cross-tabulation and test statistics was done where applicable. Chi square was used to compare rates, ratios, and proportions while fishers' exact test was used when cells have expected values less than 5. T test was used to determine the association between the continuous variables. Inferential statistics were done using chisquare to assess and compare the relationships among the categorical variables (Independent) with the overall Dependent variable.

A binary logistic regression analysis was used to identify the determinants influencing Pulmonary Tuberculosis Among Tb Patients, using the demographic variables as independent variables and good preventive practices as the outcome variable. All the significant variables during the bivariate analysis were imputed into the logistic mod el. Adjusted odds ratio (AOR) and 95% confidence interval were also obtained. The level of significance was less than 0.05

## Results

The table 1 revealed the socio demographic variables of respondents. The mean age of cases is 43.9 years, while it is 42 years for controls. However, age does not show a statistically significant association with TB status. There are more males in both the cases (78.6%) and controls (78.6%). However, gender does not show a significant association with TB status. There are no significant differences in TB status between single, married, divorced, separated, and widowed individuals. Christianity and Islam are the two main religions in the study. Christianity shows a significant association with TB status, while Islam does not. Yoruba is the dominant ethnic group in both cases (89.8%) and controls (46.2%). Ethnicity shows a significant association with TB status, with Yoruba individuals having higher odds of TB. Individuals with no education, primary education, and secondary education have higher odds of TB compared to those with tertiary education (p = 0.026).

**Table 1:** Sociodemographic Characteristics of respondents

Variable	Cases n(%)= 108	Controls n(%) =108
**Age in years**	43.9 (60)	42 (91.3)
**Gender**		
Male	70 (78.6)	70 (78.6)
Female	38 (21.3)	38 (21.3)
**Highest level of education**		
No education	5 (4.6)	11 (12.4)
Primary education	13 (15.2)	19 (20.1)
Secondary education	34 (36.5)	46 (48.2)
Tertiary education	56 (57.2)	32 (33.7)
**Religion**		
Christian	63 (58.3)	50 (46.2)
Muslim	44 (40.74)	58 (53.7)
African traditional religion	1 (0.92	0 (0
**Ethnicity**		
Yoruba	97 (89.8)	92 (46.2)
Igbo	9 (8.3)	6 (53.7)
Hausa	2 (1.85	10 (0)
**Marital status**		
Single	36 (26.6)	63 (41.3)
Married	67 (62)	35 (25.3)
Divorced	0 (0)	3 (2.7)
Separated	4 (3.7)	4 (3.7)
Widowed	1 (0.92)	3 (3.7)
**Occupation**		
Trader	27 (25)	27 (33.3)
Civil servant	38 (35.1)	22 (66.6)
Artisan	1 (0.9)	0 (0)
Self employed	23 (21.2)	39 (36.1)
Unemployed	5 (4.6)	13 (11.8)
Student	14 (12.9)	7 (5.9)
**Household monthly income**		
≥ 30,000 naira/month	39 (36.2)	51 (48.9)
< 30,000 naira/month	69 (61.5)	57 (52.6)
**Residential area**		
Large city / urban	73 (48.6)	82 (54.6)
Small city / rural	77 (51.3)	68 (45.3)

## Discussion

This study provides explanation of the results presented in the previous chapter in relation to existing literatures. The results showed that smoking more than a pack (20 cigarettes) per day was associated with TB infection. Previous studies both in developed and developing countries have shown an increased vulnerability of smokers to the infection and development of TB, most probably owing to patho-physiological changes in the lungs induced by chronic smoking 14, 15.

According to a study Bronchoalveolar macrophages among smokers contain high levels of iron implicated in promoting the growth of M. tuberculosis. Smoking 1 pack of cigarettes is equivalent to inhaling 1.1 µg of iron posit this. In this study, patients smoking more than one pack per day were twice as likely to have TB as those who smoke less than one pack per day. Smoking is great public health issue in Nigeria, even in hospitalized patients. Our results might reinforce the need to devise effective strategies for counseling TB patients and their relatives about quitting smoking^16^.

The present study found no association between alcohol consumption and TB, which differs from study who concluded that alcohol had a stronger association with tuberculosis. Ruffino-Neto, several decades ago, investigated the role of alcohol consumption and smoking in the risk of developing tuberculosis and found that smoking was associated with tuberculosis only in the group of drinkers. However, alcoholics should also be counseled effectively to wean them away from these unhealthy behaviors and minimize the risk of developing the disease^17^.

The low household income (<30,000 naira per month, approximately 43 US Dollars) appears to be a risk factor for developing TB in the study. Measures of poor living conditions like low family income, poor household equipment and malnutrition have been found to be associated with an increased risk of developing tuberculosis. This agrees with a study where Poverty undoubtedly contributes to the incidence of tuberculosis through increased progression from infection to disease due to poor diet or stress, and greater difficulties in using health services^18^. In Osun state, a study has also observed that poverty is not only a risk factor for TB, but also the single greatest barrier that prevents people under treatment from becoming cured. This is especially true when one's household responsibilities do not allow them to submit to the long hospitalization periods that are standard in Osun state treatment, particularly for drug-resistant TB^19^.

Lower level of education and poor knowledge about TB appeared to favor the development of the disease. TB more strongly affects disadvantaged population groups. However, illiterate individuals need to be focused on a priority basis for educational interventions regarding the disease. WHO also recognizes the importance of tuberculosis-related knowledge, attitude and practice surveys in advocacy, communication and social mobilization strategy planning 20.

The findings are also consistent with a study that found a strong association between a reported presence of TB patient in the family and the development of TB. This risk increases with the number of persons in the household having had TB in the past, due to a higher chance of getting the TB bacillus via their respiratory tract. This result supports the recommendation of the Tuberculosis Control Cluster, which recommends the special care of persons who have had close contact with a TB patient 21.

The results suggest that comorbid conditions like diabetes and HIV, as well as living conditions, such as the type of house, number of rooms, length of residence, and roof material, are significant factors influencing the development of TB among the study participants. However, these are univariate analyses, and a multivariate analysis is needed to determine which factors are independently associated with TB when other factors are controlled.

## Conclusion

The results indicate that certain lifestyle and housing characteristics might be associated with the risk of developing active pulmonary tuberculosis, emphasizing the potential role of environmental factors in TB transmission and development. Significant differences are found between cases and controls in several factors including gender, ethnicity, education level, household monthly income, number of cigarettes smoked per day, diabetes medication status, and HIV status. These factors may play a role in the development of pulmonary tuberculosis, but multivariable analyses are needed to further explore these associations while controlling for potential confounding variables. In conclusion, the development of pulmonary tuberculosis is influenced by a range of factors, including socio-economic status, lifestyle habits, comorbid conditions, and living conditions.

Therefore, comprehensive and integrated approaches are needed to effectively tackle TB. These approaches should not only address the medical aspects of TB control but also the broader socio-economic and environmental factors that contribute to TB development. The findings of this study provide valuable insights that can inform such comprehensive and integrated TB control efforts in Osun State, Nigeria.

## Study Limitation

This was a descriptive cross-sectional study design and social desirability bias could have been introduced because of the use of the interviewer-administered type of data collection tool. However, the research assistants were well trained to minimize this bias.

## Figures and Tables

**Table 2:** Assessment of Respondents Life style characteristics

Variables	Cases	Controls	OR(95% CI)	P-value
	n(%)=108	n(%)=108		
**Do you smoke?**				
Yes	4 (3.7)	15 (12.5)	-	0.325
No	104 (98.9)	93 (30)	0.78 (0.48-1.27)	
				
**If yes, how many cigarette per day?**				
1 pack/day	2 (1.85)	10 (9.2)	-	0.009
2 pack/day	2 (1.85)	4 (3.1)	1.46 (1.10-1.94)	
				
**Do you drink Alcohol?**				
Yes	42 (39.2)	53 (49.1)	-	0.412
No	66 (62.3)	53 (49.1)	0.82 (0.52-1.30)	
				
**If yes, how many bottles per day?**				
1	25 (18.9)	33 (30.5)	-	0.122
2	18 (11.5)	18 (16.6)	1.61 (0.87-2.95)	
3	0	4 (3.7)		

OR = odds ratio; CI = confidence interval.

* P ≤ 0.05 (significance level).

**Table 3:** Occurrence of Co-morbid conditions among respondents

Variables	Cases n(%)=108	Controls n(%) =108	OR (95% CI)	P-value
**Are you diabetic?**				
Yes	53 (49.7)	105 (99.3)	1.58 (1.23-2.02)	0.005
No	97 (95)	45 (39)	-	
				
**If yes, are currently on drugs?**				
Un-vaccinated	46 (41.6)	69 (64)	0.84 (0.3-1.79)	0.49
Vaccinated	51 (34)	36 (24)		
				
**HIVstatus**				
Positive	66 (61.2)	59 (52.3)	1.69 (1.37-2.08)	0.001
Negative	84 (79.9)	91 (89.9)	-	

**Table 4:** Housing Status among respondents

Variables	Cases	Controls	OR (95% CI)	P-value
	n(%)= 108	n(%) =108		
**Type of House**				
One room	10 (9.2)	21 (19.4)	1.67 (1.30-2.14)	0.005
Self-contain	27 (25)	15 (13.8)		
A flat	18 (16.6)	26 (24.1)		
2 bedrooms flat	39 (36.1)	32 (29.6)		
3 bedrooms flat	11 (10.1)	14 (12.9)		
Duplex	3 (2.8)	0 (0)		
**Total number of people in the household**				
< 4 people	67(61.8)	68(63)	0.58 (0.28-1.21)	- 0.145
> 4 people	41(37.7)	40(36.9)		
**Total number of rooms in the house**				
< 4 rooms	58(57.2)	57(52.6)	1.46 (1.10-1.94)	- 0.27
> 4 rooms	50(42.4)	51(47)		
**House ownership?**				
Owned	41 (37.9)	33 (30.5)	1.49 (1.09-2.04)	0.006
Rented	67 (62.0)	75 (69.4)	-	
**The roof is made up of?**				
Aluminum	0 (0)	2 (1.8)	-	
Asbestos sheet	97 (89.8)	99 (91.6)	1.96 (1.55-2.47)	0.001
PVC sheet	11 (10.1)	7 (6.4)	-	

OR = odds ratio; CI = confidence interval.

* P ≤ 0.05 (significance level).

## Data Availability

All information gathered were kept confidentially. Participants were identified using serial numbers; names of respondents were not requeste

## Right of decline/withdraw from study

Respondents were told that participation is voluntary and they would not suffer any consequences if they chose not to participate in the study.
